# Impacts of growth form and phylogenetic relatedness on seed germination: A large‐scale analysis of a subtropical regional flora

**DOI:** 10.1002/ece3.7132

**Published:** 2021-01-07

**Authors:** JuHong Wang, GeXi Xu, Wen Chen, YanBo Ma, Wei Qi, ChunHui Zhang, XianLiang Cui

**Affiliations:** ^1^ College of Food Technology and Life Science Hanshan Normal University Chaozhou China; ^2^ Key Laboratory of Forest Ecology and Environment of National Forestry and Grassland Administration Research Institute of Forest Ecology, Environment and Protection Chinese Academy of Forestry Beijing China; ^3^ College of Geography and Tourism Management Hanshan Normal University Chaozhou China; ^4^ College of Mathematics and Statistics Hanshan Normal University Chaozhou China; ^5^ State Key Laboratory of Grassland Agroecosystems School of Life Sciences Lanzhou University Lanzhou China; ^6^ State Key Laboratory of Plateau Ecology and Agriculture Qinghai University Xining China; ^7^ College of Biology and Chemistry Puer University Puer China

**Keywords:** China, controlled experiment, phylogenetic signal, seed germination response, subtropical flora

## Abstract

Plant regeneration strategy plays a critical role in species survival and can be used as a proxy for the evolutionary response of species to climate change. However, information on the effects of key plant traits and phylogenetic relatedness on seed germination is limited at large regional scales that vary in climate. To test the hypotheses that phylogenetic niche conservatism plays a critical force in shaping seed ecophysiological traits across species, and also drives their response to climatic fluctuation, we conducted a controlled experiment on seed germination and determined the percentage and rate of germination for 249 species in subtropical China under two temperature regimes (i.e., daily 25°C; daily alternating 25/15°C for each 12 hr). Germination was low with a skewed distribution (mean = 38.9% at 25°C, and 43.3% at 25/15°C). One fifth of the species had low (<10%) and slow (4–30 days) germination, and only a few (8%) species had a high (>80%) and rapid (1.2–6.6 days) germination. All studied plant traits (including germination responses) showed a significant phylogenetic signal, with an exception of seed germination percentage under the alternating temperature scenario. Generalized linear models (GLMs) and phylogenetic generalized estimation equations (GEEs) demonstrated that growth form and seed dispersal mode were strong drivers of germination. Our experimental study highlights that integrating plant key traits and phylogeny is critical to predicting seed germination response to future climate change.

## Graphical abstract

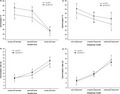

## 1 INTRODUCTION

The reproduction and regeneration strategies of plants have long been of interest to evolutionary ecologists. In particular, the pattern and timing of seed germination, as a crucial life‐history strategy (Willis et al., 2014), can determine subsequent plant performance (Bolmgren & Eriksson, 2015; Donohue et al., 2005). Germination phenology is also thought to play a significant role in community composition and succession (Schütz, 2002) and may even contribute to the abundance and distribution of species (Chambers et al., 1990; Liu et al., 2013). In general, a rapid germination rate enables seedlings to start developing as soon as conditions become favorable for germination, and thereby to maximize the growing period and minimize seed predation risk (Willis et al., 2014). In contrast, in species with seeds of various degrees of dormancy, plants can distribute their offspring across time and bet‐hedge against unpredictable and variable environments (Clauss & Venable, 2000; Poisot et al., 2011; Venable, 2007).

In seasonal environments, germinating early in the growing season should permit a plant to grow longer and acquire more resources for reproduction than a plant from a late‐germinating seed. However, early germination also carries the risk that seedlings will be exposed to adverse environmental conditions, such as low temperatures or frost (Baskin & Baskin, 2014). The germination behavior of species among and within plant communities varies considerably as a function of environmental cues (Grime et al., 1981; Olff et al., 1994; Villa‐Reyes & Barrera, 2016), and this variation may mitigate competition or allow seedlings to develop under different seasonal environmental conditions (Huang et al., 2016). Thus, there is much potential for natural selection to act on germination, which would optimize regeneration strategies with respect to environmental conditions.

Several broad comparative surveys have shown that species' seed germination is related to environmental factors (Westoby, 1981), including temperature (Baskin & Baskin, 2014), rainfall (Gutterman, 2000), light (Kyereh et al., 1999), and altitude (Meyer & Kitchen, 1994), and also life‐history traits such as seed mass (Garwood, 1983; Guo et al., 2000; Shipley & Parent, 1991), dispersal mode (Willson & Traveset, 2000) and growth form (Figueroa, 2003).

These functional traits may be constrained by the phylogenetic history of each species (Bu et al., 2007; Figueroa & Armesto, 2001). The relationship between phylogenetic relatedness and ecological similarity among species can be quantified using phylogenetic signal, based on phylogenetic niche conservatism (PNC) theory (Losos, 2008). The phylogenetic signal reflects the tendency of closely related species to have more similar niches and functional traits than species randomly drawn from the phylogenetic tree (Blomberg et al., 2003). Thus, the phylogenetic signal represents the retention of niche‐related traits within lineages over macroevolutionary time, despite speciation events (Crisp & Cook, 2012; Wiens et al., 2010). Phylogenetic analysis has revealed that many (possibly the majority of) lineages studied show evidence for conservatism of dominant ecological characters. In particular, reproductive strategies such as germination are affected by phylogenetic constraints and evolutionary allometries that limit divergence (Kochmer & Handel, 1986; Mazer, 1989; Mckitrick, 1993; Zhang et al., 2014). Therefore, investigating the phylogenetic relatedness of seed traits and interactions between plant traits at the community level or larger scale flora can provide important insights into the role of natural selection in shaping seed traits and community assembly (Webb et al., 2002).

In this study, we examined the seed germination characteristics of 249 species in southern China. We hypothesized that phylogenetic niche conservatism plays a critical force in shaping the ecophysiological traits of seeds, as well as driving correlations between plant traits due to potential climatic fluctuation. To answer these questions, we tested for a phylogenetic signal in each plant trait. Then we examined how germination (i.e., percentage and rate) varied according to these traits under two temperature scenarios, using both generalized linear models and phylogenetic generalized estimation equations.

## 2 MATERIALS AND METHODS

### 2.1 Study region

We studied a total of 249 angiosperm species from two regions in China: 91 species from southeastern China (Chaozhou, Guangdong Province) (114°53′E–117°08′E, 22°31′N–24°15′N) and 158 species from mid‐south China (Changsha, Hunan Province) (111°53′–114°15′E, 27°51′–28°41′N). These species are widely distributed on the edges of farmland, weed fields, roadsides, and wasteland, and are the most abundant taxa in their respective zone. The average annual temperature of Chaozhou is 21°C, with a mean monthly maximum temperature of 28°C in July and monthly minimum temperature of 7–15°C in January (Wu et al., 2014). The annual rainfall varies between 1,300 and 2,400 mm, with the wet season extending from April to September and the dry season from October to January (Chen et al., 1997). The average annual temperature of Changsha is 17.2°C, with a mean monthly maximum temperature of 29.4°C in July, and a mean monthly minimum temperature of 4.7°C in January. Mean annual rainfall is 1,360 mm. The two regions both belong to a subtropical humid monsoon climate.

### 2.2 Seed collection

Mature seeds of the 249 common species were collected between August and November in 2015 and 2016. Seeds were collected from one to three populations for each species with at least 20–30 randomly selected individuals. All seeds of each species were pooled, air‐dried at room temperature for 1–2 days, and then stored in dry conditions at 4°C (mean = 15 days, to avoid effects of low temperature and dry storage on the physiology of the seeds) until the onset of germination tests. Seed mass of each species was determined by weighing three replicates of 100 seeds. Seed vigor was assessed with the tetrazolium test before initiation of experiments (Hendry & Grime, 1993). Three replicates of 50 seeds of each species were placed on moist filter paper at room temperature for 24 hr and then sliced along the longitudinal axis with a scalpel. Both seed sections were incubated in a 0.1% aqueous solution of tetrazolium chloride for 24 hr at 25°C in darkness. Seeds with a strong red‐stained embryo were considered to be viable (Baskin & Baskin, 2014).

### 2.3 Germination test

The final germination percentage and rate (speed) of each species were determined under laboratory conditions. For each species, three replicates of 50 seeds were placed in 9‐cm‐diameter Petri dishes on two layers of filter paper moistened with distilled water. Seeds of each species were incubated under two temperature regimes: either a constant temperature of daily 25°C (the mean high temperature during the spring germination period), or alternating temperatures of 25 and 15°C for each 12 hr (the high and low mean annual temperatures). Seeds were in the same daily light‐dark cycle in each temperature regime. Light was provided by fluorescent tubes (PPFD, 25–30 μmol/m^−2^ s^−1^ at seed level) for 12 hr each day (i.e., during the high‐temperature phase of the alternating temperature regime). Seed germination was monitored every 24 hr for 30 days, and a seed was considered to be germinated when the radical was visible to the naked eye. Germinated seeds were counted and then discarded. At the end of the germination tests, viability of non‐germinated seeds was determined by opening each seed with a needle to check if the embryo was firm and white (viable) or soft and gray (non‐viable) (Baskin & Baskin, 2014).

Two germination indices were used in this study (Wang et al., 2012). Germination percentage (GP) was calculated as GP = GN × 100/SN, where GP is the final germination percentage (%), GN is the total number of germinated seeds, and SN is the total number of seeds tested. The mean value of the three replicates was calculated. Germination rate (GR) was calculated as GR = ∑(*G_i_* × *i*)/∑*G_i_*, where *i* is number of days between seed sowing (day 0) and seed germination, and *G_i_* is the number of seeds germinated on day *i*. GR corresponds to the mean germination time of the fraction of seeds that germinated.

### 2.4 Plant traits

Four traits were selected to capture information on various aspects of plant ecological strategy (traits are listed in Table 1). Seed mass and seed dispersal syndrome provide information about a species' reproductive strategy (Westoby et al., 1992; Wright et al., 2010). Maximum plant height and growth form provide crucial information about resource acquisition, competitive ability, and life‐history strategy (Falster & Westoby, 2005). Plant seed dispersal mode (unassisted, vertebrate, wind), growth form (annual or biennial herb, perennial herb, shrub, or tree), and height were extracted from the *Flora of China* (http://www.iplant.cn/foc/).

**TABLE 1:** Information on the plant traits for the 249 study species

Trait	Species (*n*)	Species (%)	Trait	Species (*n*)	Species (%)
Growth form:			Mass of a single seed (mg):		
Annual or biennial herb	88	35.3	<0.5	82	32.9
Perennial herb	94	37.8	0.5–1.0	24	9.6
Shrub or tree	67	26.9	1.0–1.5	10	4.0
Plant height:			1.5–2.0	16	6.4
<50 cm	22	8.8	2.0–3.0	31	12.4
50–99 cm	61	24.5	3.0–5.0	20	8.0
100–149 cm	58	23.4	5.0–10	20	8.0
150–199 cm	24	9.6	10–20	24	9.6
200–249 cm	33	13.3	>20	22	8.8
250–300 cm	23	9.2			
>300 cm	28	11.2			
Dispersal mode:					
Wind	47	18.9			
Unassisted	167	67.0			
Vertebrate	35	14.1			

### 2.5 Statistical analysis

We reconstructed a phylogenetic tree representing the 249 studied angiosperm species based on Angiosperm Phylogeny Group III (2009), using the Phylomatic platform (version 3, http://phylodiversity.net/phylomatic/) with the “zanne2014” as a stored tree (Zanne et al., 2014). We randomly resolved the polytomies on this phylogenetic tree using the *multi2di* function within the *ape* package (Swenson, 2014). An ultrametric phylogenetic tree was obtained for further analysis. Given the uncertainty caused by the random‐split polytomies, we resolved the polytomies 10 times and reran the corresponding analysis each time to examine any variation of results to this uncertainty (Losos, 1994).

To quantify the degree to which the phylogenetic tree estimated the trait similarity of species, we quantified the phylogenetic signal for continuous data (germination rate, seed mass, and plant height) using the function *phylosig* within the package *phytools* (Revell, 2012). The phylogenetic signal was quantified using Pagel's *λ* statistic (Pagel, 1999). When *λ* = 0, the trait is more dissimilar among related taxa than expected by chance; when *λ* > 0 (maximum *λ* = 1) with a significant phylogenetic signal, the evolution of the trait is more conservative compared to stochastic expectations, that is, more similar among related taxa (Pagel, 1999; Vandelook et al., 2017). To test for the significance of *λ*, the phylogenetic tips (i.e., taxa) were randomly shuffled on the phylogeny, then the functional traits were arrayed on it specifically (Yang et al., 2014). This randomization was carried out 999 times to generate a null distribution from which a *p‐*value could be calculated.

For germination percentage (a binomial variable, of the number of germinated and ungerminated seeds), we calculated the phylogenetic *D* statistic using the function *phylo.d* within the package *caper* (Fritz & Purvis, 2010). If *D* ≤ 0, the trait evolved highly conservatively across the tips (i.e., Brownian motion), whereas if *D* ≥ 1, the trait is over‐dispersed and evolved randomly among the taxa (Vandelook et al., 2017). The significance of *D* was tested under 1,000 random permutations of the phylogenetic tips. In addition, we calculated the *δ* statistic, as proposed by Borges et al. (2019), to evaluate the effects of phylogeny on a categorical trait's evolution across species. The *δ* value increases when the trait evolved conservatively and decreases when the trait evolved independently across species. Thus, a higher *δ* value indicates a stronger phylogenetic signal between a specific categorical trait and the phylogenetic tree.

To test the response of seed germination to plant traits under distinct temperature conditions, we fitted generalized linear models (GLMs) with a logit link function and binomial distribution for germination percentage and an identity link function and gaussian distribution for germination rate (Salazar et al., 2018). In addition, phylogenetic generalized estimating equations (GEEs; extensions of GLMs) were used to evaluate the potential effects of seed mass, plant height, dispersal mode, and growth form on germination response on the basis of phylogeny (Paradis & Claude, 2002). Growth form (annual or biennial herb = 1, perennial herb = 2, shrub or tree = 3) and dispersal mode (unassisted = 1, vertebrate = 2, wind = 3) were included as categorical variables.

All statistical analysis was performed using the R environment for statistical computing (R Development Core Team, 2015). Data were plotted using the software Origin 9.1 (Origin Lab: https://www.originlab.com).

## 3 RESULTS

### 3.1 Germination characteristics

Germination percentage (GP) of the 249 study species ranged from 0% to 98% (mean = 38.9%; median = 33%) at 25°C and 0 to 100% (mean = 43.3%; median = 38%) at 25/15°C. A total of 95 species (38.2%) reached ≥ 50% germination at 25°C and 112 species (44.5%) at 25/15°C. A total of 70 species (28.1%) had < 10% germination at 25°C and 59 species (23.7%) at 25/15°C.

Germination rate (GR) of the 249 species ranged from 1.2 to 28.0 days (mean = 9.77 days) at 25°C and 1.17 to 30 days (mean = 9.99) at 25/15°C. A total of 17 species (at 25°C) and 20 (at 25/15°C) germinated within 3 days; and 27 species (25°C) and 26 species (25°C) exceeded 20 days.

Seeds of 20 species (8%) had high (>80%) and rapid (1.2–6.6 days) germination under both temperature regimes (Appendix A). Seeds of 49 species (19.6%) had low (<10%) and slow (4–30 days) germination, except for *Malva sinensis* (No. 21), *Tagetes patula* (No. 29), and *Amaranthus tricolor* (No. 44) (Appendix B).

**FIGURE 1:**
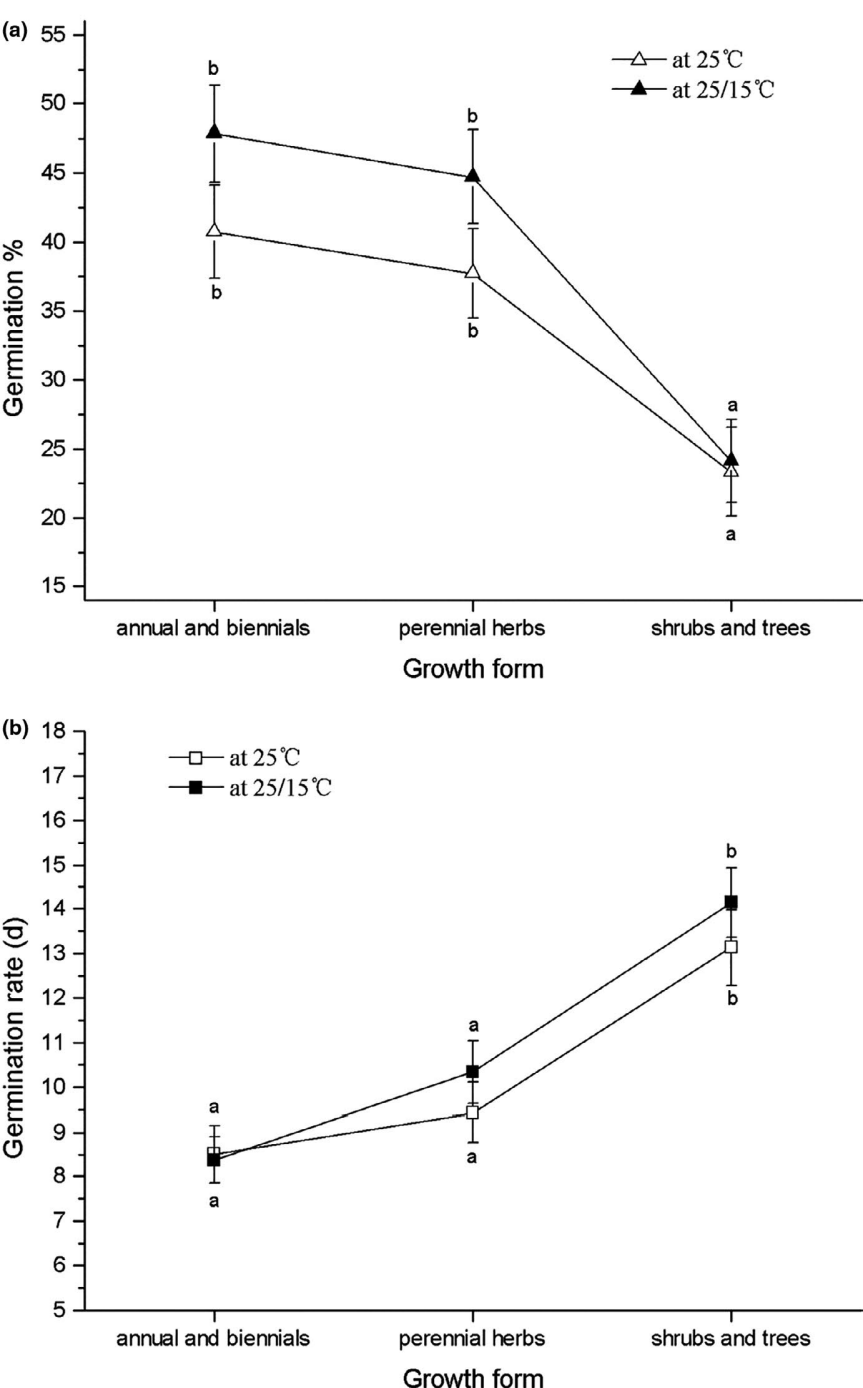
Differences in germination percentage (a) and rate (b) between species with different growth forms

**FIGURE 2:**
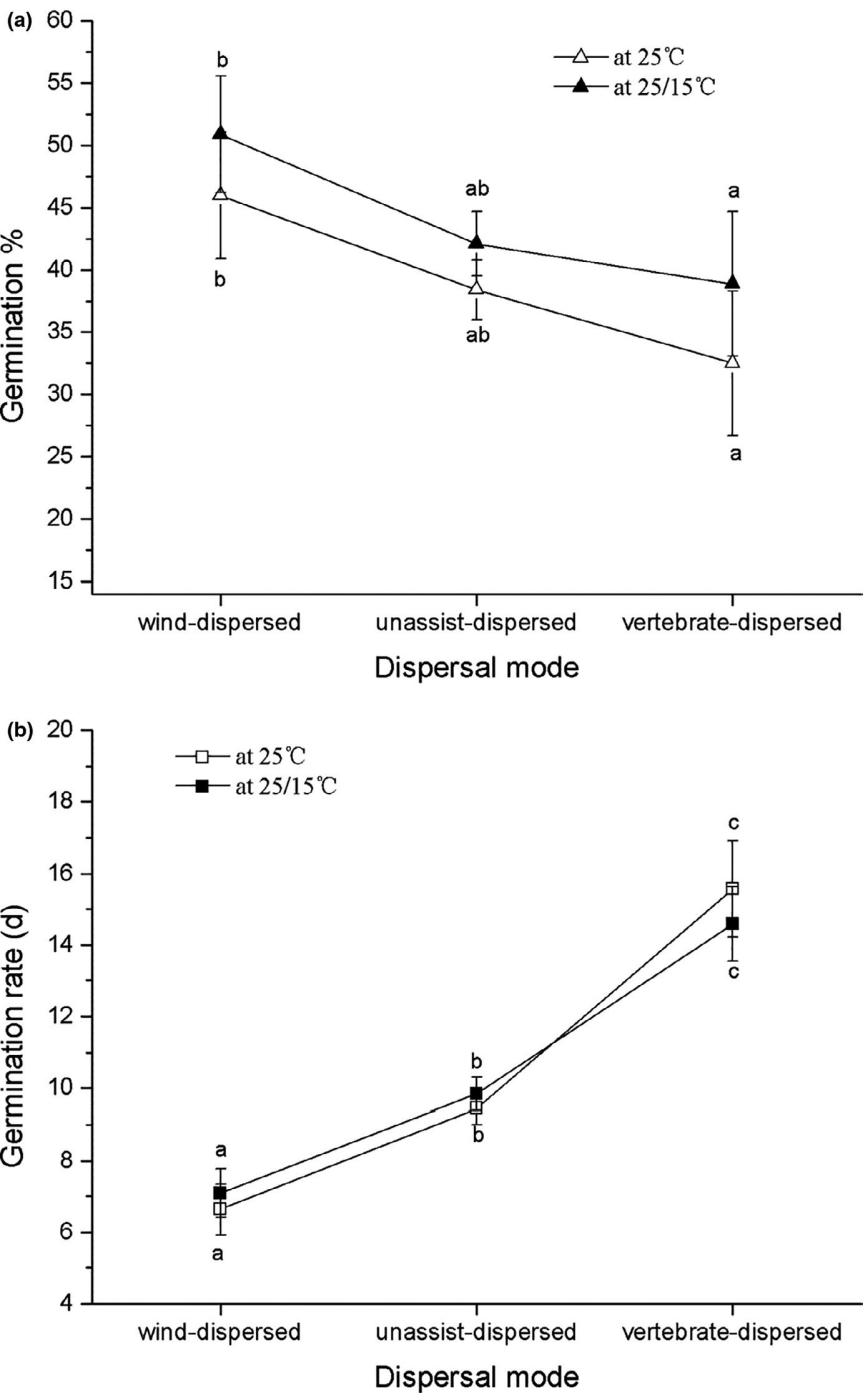
Difference in germination percentage (a) and rate (b) between species with different dispersal modes

Seeds of annual or biennial herbs (mean GP = 45.5% and 52.6%, at 25°C and 25/15°C, respectively the same below; mean GR = 7.8 and 8.2 days) and perennial herbs (mean GP = 41.1% and 45.8%; mean GT = 9.1 and 9.8 days) had higher and faster germination than shrubs and trees (mean GP = 27.5%; mean GR = 12.7 and 13 days) (Figure 1).Wind‐dispersed seeds had higher germination (mean GP = 45.9% and 50.9%) and especially more rapid germination (mean GR = 6.6 and 7.1 days) than unassisted dispersal (mean GP = 38.5% and 42.1%; mean GR = 9.4 and 9.8 days) and vertebrate‐dispersed seeds (mean GP = 32.5% and 38.8%; mean GR = 14.6, and 15.6 days) (Figure 2).

### 3.2 Phylogenetic signal

Seed mass (Pagel's *λ* = 1.00) and plant height (Pagel's *λ* = 0.98) showed remarkably high phylogenetic signal. Seed germination rate (GR) had a moderately high and significant phylogenetic signal under both temperature regimes (*λ* = 0.46 at 25°C, and *λ* = 0.67 at 25/15°C). The phylogenetic signal in germination percentage (GP) was conservative under both temperature treatments (*D* = 0.89 at 25°C treatment, and *D* = 0.93 at alternative 25/15°C treatment), but was only significant at the constant temperature treatment. In addition, when compared with growth form (*δ* = 0.00), seed dispersal mode showed a relatively high phylogenetic signal (*δ* = 9.07; Table 2).

**TABLE 2:** Phylogenetic signals for continuous traits (Pagel's *λ*), binomial traits (*D* statistic) and categorial traits (*δ* value) in this analysis

Trait	Data type	*λ*‐value	*p*‐value	*D*‐value	*p*‐value	*δ*‐value
Seed mass (g)	Continuous	1.00	<0.01	—	—	—
Height (m)	Continuous	0.98	<0.01	—	—	—
Germination rate at daily 25°C	Continuous	0.46	<0.01	—	—	—
Germination rate at alternating daily 25/15°C	Continuous	0.67	<0.01	—	—	—
Germination percentage at daily 25°C	Binomial	—	—	0.89	0.03	—
Germination percentage at alternating daily 25/15°C	Binomial	—	—	0.93	0.12	—
Growth form (annual, perennial, woody)	Categorical	—	—	—	—	1.05
Dispersal mode (wind, unassisted, vertebrate)	Categorical	—	—	—	—	9.07

### 3.3 Effect of plant traits on germination response

The generalized linear models (GLMs; binomial distribution) showed that seed germination percentage (GP) under the two temperature regimes was significantly different only for plant growth form. Specifically, GP was much lower in shrubs and trees compared to herbs.

Seed germination rate (GR) varied as a function of only dispersal mode and growth form at 25°C, but varied in response to all traits in the alternating 25/15°C temperature regime. Comparing with wind‐dispersed seeds, unassisted dispersal had a higher GR, followed by vertebrate dispersal. Similarly, germination rate was slower for shrubs and trees compared to herbs. Increasing seed mass and plant height also delayed the germination rate moderately (Table 3).

**TABLE 3:** Results of generalized linear models (GLMs) and the phylogenetic generalized estimating equations (GEEs) depicting the potential effects of key plant traits on germination responses at varied temperature conditions

Response variables	Predictor variables	Across‐species GLMs				Phylogenetic GEEs			
		*Coef*.	*SE*	*z‐*value	*p*	*Coef*.	*SE*	*t*‐value	*p*
Germination percentage under temperature treatment of daily 25°C	°C Seed mass	–0.00	0.01	–0.56	0.58	0.00	0.01	0.13	0.90
	Height	0.00	0.00	0.75	0.45	0.00	0.00	0.16	0.87
	Dispersal mode (Vertebrate)	–0.41	0.43	–0.95	0.34	–0.58	0.39	–1.49	0.14
	Dispersal mode (Wind)	–0.24	0.40	–0.59	0.56	**0.79**	**0.39**	**2.04**	**0.05**
	Growth form (Perennial herb)	–0.39	0.36	–1.08	0.28	**–2.49**	**0.35**	**–7.02**	**<0.01**
	Growth form (Shrub and tree)	**–0.99**	**0.44**	**–2.24**	**0.03**	**–2.23**	**0.37**	**–6.10**	**<0.01**
Germination percentage under temperature treatment of daily alternating 25/15°C for each 12 hr	Seed mass	0.01	0.01	–1.79	0.07	–0.01	0.01	–1.14	0.26
	Height	0.00	0.00	0.15	0.88	–0.00	0.00	–1.64	0.11
	Dispersal mode (Vertebrate)	0.09	0.44	–0.19	0.85	–0.64	0.41	–1.58	0.12
	Dispersal mode (Wind)	0.24	0.47	0.51	0.61	0.51	0.46	1.12	0.27
	Growth form (Perennial herb)	–0.30	0.40	–0.75	0.45	**–3.09**	**0.39**	**–7.96**	**<0.01**
	Growth form (Shrub and tree)	**–1.00**	**0.46**	**–2.16**	**0.03**	**–2.47**	**0.39**	**–6.38**	**<0.01**
Germination rate under temperature treatment of daily 25°C	Seed mass	0.00	0.00	1.92	0.06	**0.00**	**0.00**	**3.92**	**<0.01**
	Height	0.00	0.00	1.46	0.15	0.00	0.00	0.63	0.53
	Dispersal mode (Vertebrate)	**0.43**	**0.06**	**7.70**	**<0.01**	0.14	0.10	1.52	0.13
	Dispersal mode (Wind)	**–0.30**	**0.06**	**–4.64**	**<0.01**	**–0.78**	**0.30**	**–2.61**	**0.01**
	Growth form (Perennial herb)	0.02	0.05	0.46	0.65	**0.29**	**0.05**	**5.31**	**<0.01**
	Growth form (Shrub and tree)	**0.14**	**0.06**	**2.33**	**0.02**	0.11	0.06	1.68	0.10
Germination rate under temperature treatment of daily alternating 25/15°C for each 12 hr	Seed mass	**0.002**	**0.00**	**2.48**	**0.01**	**0.004**	**0.00**	**5.69**	**<0.01**
	Height	**0.00**	**0.00**	**4.39**	**<0.01**	**0.00**	**0.00**	**2.31**	**0.02**
	Dispersal mode (Vertebrate)	**0.30**	**0.06**	**5.46**	**<0.01**	0.11	0.06	1.78	0.08
	Dispersal mode (Wind)	**–0.21**	**0.06**	**–3.42**	**<0.01**	**–0.40**	**0.16**	**–2.44**	**0.02**
	Growth form (Perennial herb)	**0.16**	**0.05**	**3.14**	**<0.01**	**0.23**	**0.03**	**7.00**	**<0.01**
	Growth form (Shrub and tree)	**0.24**	**0.06**	**3.92**	**<0.01**	**0.22**	**0.05**	**4.73**	**<0.01**

Note

*Coef*. and *SE* indicates coefficient and standard error in GLM or GEE regression model. Significant predictor variables in the models are showed in bold.

Phylogenetic relatedness increased the role of growth form in explaining variation of germination percentage under both temperature regimes. The germination percentage was greater in annual or biennial herbs compared to perennials. Additionally, species with wind‐dispersed seeds had a higher germination percentage compared to those with unassisted or vertebrate dispersal at 25°C. For germination rate, the phylogenetic GEEs showed that variability of germination rate was sensitive to dispersal mode and growth form, and to some extent seed mass and height, especially at 25/15°C. Wind‐dispersed seeds germinated faster than those unassisted or assisted by vertebrates. However, the germination rate was slower in shrubs and trees compared to annual, biennial, and perennial herbs (Table 3).

## 4 DISCUSSION

### 4.1 Seed germination characteristics

Germination strategy is of great ecological and evolutionary interest (Donohue, 2002). Numerous studies have shown that species differ considerably in germination strategy in response to environmental cues (Fenner & Thompson, 2005; Grime et al., 1981). In general, high germination percentage may give a high colonization capacity, but seed dormancy prevents seeds from germinating rapidly after maturation, which is regarded as a mechanism allowing plants to survive in temporally variable and unpredictable environments (Clauss & Venable, 2000). Rapid germination may gain a competitive advantage in favorable conditions (Jiménez‐Alfaro et al., 2016), but slow germination (which may fail to germinate during these short favorable periods) could provide a benefit in the long run, if seeds remain viable while the seedlings of rapid germinators are killed by unsuitable environmental conditions (Grubb, 1977).

In our study of 249 species, germination was low and/or slow, inconsistent with similar data from tropical forest species in Ghana (Kyereh et al., 1999) and Panama (Sautu et al., 2006) that was normally distributed, and inconsistent with the bimodal data from 69 arid and semi‐arid zone species (Wang et al., 2009). Our study site was located in southern China, where minimum winter temperatures range from 7 to 15°C in Chaozhou and 4 to 7°C in Changsha, with extreme minimum temperatures of −0.5°C and −9°C, respectively (Wu et al., 2014). Low temperatures in spring (e.g., <10–20°C for a period of time) may result in the rotting and decomposition of seedlings and be unfavorable for establishment (Ye, 1986). Thus, for species in subtropical regions, low temperatures may limit the survival and establishment of seedlings (Hacker & Ratcliff, 1989). We suggest that low‐and‐slow seed germination may be an important strategy for subtropical species, which would avoid seedling establishment under unfavorable conditions, ensuring that seeds germinate in sites across time and space (Grime, 2006).

In our study, 8% of species had a germination percentage > 80% and a germination rate of < 6.6 days (e.g., *Galinsoga parviflora*, *Spilanthes paniculata, Ageratum houstonianum*, *Bidens pilosa*, *Siegesbeckia orientalis*, *Ixeridium dentatum*, *Pluchea sagittalis*, *Dendranthema indicum*, Asteraceae; *Cymbopogon goeringii* and *Digitaria sanguinalis,* Poaceae; and *Hyptis suaveolens, Perilla frutescens*, Lamiaceae), reflecting an opportunistic *r*‐strategy to ensure rapid reproductive and establishment, and further to spread their population under favorable conditions (Gutterman, 2000).

### 4.2 Phylogenetic correlates of plant traits

Phylogenetic signal represents the tendency for closely related species to resemble each other more than less related taxa, as the result of shared evolutionary history (Borges et al., 2019). As such, the functional traits of closely related species are often phylogenetically conserved (Blomberg et al., 2003; Yang et al., 2014). As was expected, almost all the traits in our study species had a significant phylogenetic signal (Table 2). Recent multivariate investigations of community assembly have often found a significant pattern of phylogenetic niche conservatism (Kooyman et al., 2011; Leibold et al., 2010). For example, by testing patterns of community assembly in tropical and subtropical rain forests across regional to continental scales, Kooyman et al. (2011) found that two latitudinal regions (tropics and subtropics) have a shared evolutionary and biogeographic history.

The most surprising result of our study was that the phylogenetic signal in germination percentage was conservative under both temperature treatments, but was only significant at the constant temperature treatment. Numerous ecological and evolutionary studies have shown that temperature is a major determinant of many ecological traits at a global scale: temperature affects metabolic rates, generation time, growth, tolerance thresholds (Chen & Stillman, 2012), seed mass (Murray et al., 2004), and germination (Baskin & Baskin, 2014; Liu et al., 2013; Moles et al., 2014). In particular, germination of some species may occur in response to fluctuating temperatures, regarded as a mechanism by which seeds detect either gaps in vegetation canopies or their depth of burial in soil, as illustrated by 36% of species in a grassland community that require alternating temperatures to germinate (Liu et al., 2013). Therefore, changes in germination under stochastic conditions such as fluctuating temperatures may reflect the outcome of evolutionary processes responding to environmental constraints (Kattge et al., 2011).

### 4.3 Growth form correlates of plant traits

The results from the GLMs and phylogenetic GEEs (Table 3) revealed that seed germination percentage and rate had strong correlations with growth form and dispersal mode. Growth form is a key intrinsic property of plants (Broennimann et al., 2006), affecting survivorship (Chu et al., 2014). According to the CSR theory (Grime, 2001), each growth form has a wide range of strategies across the tolerance–competition trade‐off. Trees have a higher competitive ability (C) and are less stress‐tolerant (S) than herbs, whereas herbs also may have a ruderal (R) strategy (Grime, 2001). Ultimately, these strategies relate to stress tolerance and to regimes of resource availability and disturbance (Chapin et al., 1993). Annual and/or biennial species with short life spans and that reproduce by seeds tend to allocate their resources to the next generations, for example, higher germination percentage and earlier germination time (R strategy). However, most perennial herbs, shrubs, and trees, with longer life span, reproducing by either seeds or vegetative propagation, may allocate more resources to growth, competitive ability, or defense (S or C strategy) (Grime, 2001). In our study, annuals and biennials had a higher germination percentage and earlier germination than either perennial herbs, and shrubs or trees. These strategies are in line with an “opportunistic” strategy of short‐lived plants versus the S or C strategy of longer‐lived wood plants in response to a variable environment. Therefore, growth form may be the main driver of seed germination evolution.

Seed dispersal is an adaptive trait to avoid natural enemies, sibling interactions, and potential limitation of the available resources near parents, as well as to increase the probability of encountering a suitable establishment site (Augspurger, 1983; Cheplick, 1993; Fenner & Thompson, 2005; Janzen, 1970). The dispersal potential of seeds with different dispersal modes varies greatly. Wind‐ and vertebrate‐dispersed seeds tend to disperse further than those with unassisted dispersal, and seeds that disperse further may have higher germination than those that do not disperse because they escape specialist pests and pathogens (Willson & Traveset, 2000). In our study, seed dispersal mode significantly influenced germination: wind‐dispersed seeds had higher and faster germination than vertebrate‐dispersed seeds and those with unassisted dispersal, which is consistent with the results of Willson and Traveset (2000) and Wang et al. (2009). These results may be partly explained by the fact that vertebrate‐dispersed seeds may need to be ingested and excreted by an animal in order to promote seed germination (Fenner & Thompson, 2005). Therefore, our results suggest that seed dispersal is also an important plant trait strongly affecting variation in seed germination.

In addition, it should also be noted that the phylogenetic GEEs showed that variability of germination rate was also sensitive to seed mass, especially at alternating 25/15°C. Seed mass is also a crucial life‐history trait determining the amount of reserves that are available to establishing seedlings (Jurado & Westoby, 1992; Paradis & Claude, 2002). Many studies have found that larger‐seed species tend to have a higher chance of seedling survival in highly competitive environments (Leishman, 2001), whereas small‐seeded species may increase their probability of survival by dispersing further from the parent plant (Hyatt et al., 2003; Norden et al., 2009). Some community‐level studies have showed that smaller seeds tend to have better germinability than larger ones, such as studies in Sheffield (Grime et al., 1981), in alpine meadow herbaceous species (Bu et al., 2007), and arid and semi‐arid species (Wang et al., 2009). Moreover, the effect of seed mass on germination was regarded as the response to light (Bu et al., 2017). In our study, species mainly occurred in habitats with frequent disturbance, such as tillage, weed fields, roadsides, and wasteland, where smaller seeds might encounter more severe selective pressure than larger ones because they have less food reserves and are more easily buried. As a result, small‐seeded species tend to have more rapid germination rate than large‐seeded ones.

It is clear from our study that phylogenetic niche conservatism plays a critical force in shaping seed traits, such as seed germination percentage and rate, across plant species in the subtropical region of southern China. In addition, germination response is driven mainly by growth form, and to a lesser extent, dispersal mode and seed mass. However, as temperature often fluctuates during the germination process, the phylogenetic conservatism of seed mass and plant height is crucial to successful germination. We suggest that a dynamic balance between growth form, phylogenetic conservatism, and environmental temperature may be key to ensuring the successful germination and establishment of seedlings.

## CONFLICT OF INTEREST

The authors declare that they have no conflict of interest.

## AUTHOR CONTRIBUTIONS

**JuHong Wang:** Investigation (equal); writing – original draft (equal); writing – review and editing (equal). **GeXi Xu:** Software (equal); writing – review and editing (equal). **Wen Chen:** Data curation (equal); investigation (equal); methodology (equal). **YanBo Ma:** Software (equal). **Wei Qi:** Formal analysis (equal). **ChunHui Zhang:** Software (equal). **XianLiang Cui:** Investigation (equal); resources (equal).

## Data Availability

Data from the germination tests and analysis are available on Dryad: https://doi.org/10.5061/dryad.7h44j0zsp. The spreadsheet contains the seed germination percentage, germination rate under two temperature regimes as well as the seed mass, dispersal mode, growth form, plant height for 249 species in southern China.

## Appendix A

**TABLE A1:** Species with a germination percentage more than 80% and their germination rates

No.	Species	Family	GP (%) 25°C	GP (%) 25/15°C	GR (days) 25°C	GR (days) 25/15°C
1	*Hyptis suaveolens*	Lamiaceae	98.00	98.67	2.50	4.37
2	*Perilla frutescens*	Lamiaceae	80.67	91.33	3.48	2.22
3	*Trifolium pratense*	Leguminosae	95.33	96.00	1.23	1.26
4	*Vicia sepium*	Leguminosae	85.33	82.67	2.39	2.52
5	*Cymbopogon goeringii*	Poaceae	96.00	96.00	1.44	2.64
6	*Digitaria sanguinalis*	Poaceae	98.00	97.33	3.48	3.46
7	*Zehneria japonica*	Cucurbitaceae	88.67	92.00	5.12	5.54
8	*Galinsoga parviflora*	Asteraceae	96.00	89.33	2.91	2.58
9	*Spilanthes paniculata*	Asteraceae	96.00	98.00	3.36	3.18
10	*Ageratum houstonianum*	Asteraceae	93.33	92.67	5.11	4.25
11	*Bidens pilosa*	Asteraceae	91.33	95.33	2.65	5.61
12	*Siegesbeckia orientalis*	Asteraceae	90.00	88.67	5.70	5.84
13	*Ixeridium dentatum*	Asteraceae	89.33	92.67	3.08	2.53
14	*Pluchea sagittalis*	Asteraceae	85.33	86.67	3.45	5.13
15	*Dendranthema indicum*	Asteraceae	82.00	85.33	3.28	4.06
16	*Rumex acetosa*	Polygonaceae	94.67	96.67	3.80	1.20
17	*Buddleja albiflora*	Loganiaceae	94.67	92.67	5.85	6.56
18	*Fimbristylis acuminata*	Cyperaceae	82.00	82.67	3.45	1.43
19	*Achyranthes bidentata*	Amaranthaceae	95.33	98.00	4.19	4.41
20	*Gonostegia neurocarpa*	Urticaceae	90.67	84.00	4.60	4.42

## Appendix B

**TABLE B1:** Species with a germination percentage less than 10% and their germination rates

No.	Species	Family	GP (%) 25°C	GP (%) 25/15°C	GR (days) 25°C	GR (days) 25/15°C
1	*Ophiopogon japonicus*	Liliaceae	7.33	8.00	21.94	23.13
2	*Mentha haplocalyx*	Lamiaceae	1.33	3.33	11.00	8.33
3	*Aeschynomene indica*	Leguminosae	7.33	9.33	6.27	4.25
4	*Bauhinia glauca*	Leguminosae	3.33	0.67	7.58	7.00
5	*Cercis chinensis*	Leguminosae	1.33	4.00	8.00	20.75
6	*Indigofera hirsuta*	Leguminosae	4.00	5.33	5.61	7.75
7	*Phyllodium pulchellum*	Leguminosae	2.67	1.33	16.11	14.00
8	*Cassia alata*	Leguminosae	2.00	2.00	7.33	12.83
9	*Desmodium heterocarpon*	Leguminosae	6.67	4.00	6.50	7.83
10	*Mimosa sepiaria*	Leguminosae	7.33	6.00	15.07	23.00
11	*Podocarpium podocarpum* var. *oxyphyllum*	Leguminosae	4.00	2.00	7.00	8.00
12	*Imperata cylindrica*	Poaceae	8.00	6.00	5.20	12.19
13	*Setaria palmifolia*	Poaceae	3.33	4.67	27.17	14.25
14	*Trichosanthes baviensis*	Cucurbitaceae	1.33	4.67	4.00	8.50
15	*Loropetalum chinense*	Hamamelidaceae	3.33	10.00	19.25	8.00
16	*Abelmoschus moschatus*	Malvaceae	6.00	6.00	5.00	5.00
17	*Abutilon indicum*	Malvaceae	2.00	4.67	17.33	21.97
18	*Corchoropsis tomentosa*	Malvaceae	2.67	3.33	4.17	7.67
19	*Helicteres angustifolia*	Malvaceae	1.33	1.33	6.33	7.33
20	*Hibiscus mutabilis*	Malvaceae	8.67	10.00	11.40	18.50
21	*Malva sinensis*	Malvaceae	1.33	1.33	10.33	2.00
22	*Melochia corchorifolia*	Malvaceae	2.67	2.67	5.50	10.67
23	*Urena lobata*	Malvaceae	2.00	3.33	14.75	9.33
24	*Ambrosia artemisiifolia*	Asteraceae	1.33	7.33	16.50	5.83
25	*Bidens frondosa*	Asteraceae	1.33	4.67	21.50	16.89
26	*Coreopsis drummondii*	Asteraceae	6.67	8.00	8.00	19.31
27	*Eclipta prostrata*	Asteraceae	2.00	3.33	18.00	6.56
28	*Farfugium japonicum*	Asteraceae	4.00	8.67	7.00	5.29
29	*Tagetes patula*	Asteraceae	4.00	6.67	3.25	1.62
30	*Brucea javanica*	Simaroubaceae	0.67	0.67	6.67	18.00
31	*Chimonanthus praecox*	Calycanthaceae	6.67	5.33	8.72	6.75
32	*Ludwigia hyssopifolia*	Onagraceae	1.33	0.67	4.00	6.00
33	*Duranta repens*	Verbenaceae	1.33	0.67	15.67	15.33
34	*Vitex negundo* var. *cannabifolia*	Verbenaceae	1.33	2.67	24.50	18.67
35	*Buddleja lindleyana*	Loganiaceae	0.67	2.00	16.00	18.83
36	*Morinda umbellata*	Rubiaceae	8.67	2.00	24.47	16.00
37	*Rosa cymosa*	Rosaceae	0.67	5.33	13.00	16.50
38	*Rosa multiflora* var. *cathayensis*	Rosaceae	0.67	2.00	26.00	8.50
39	*Spiraea salicifolia*	Rosaceae	3.33	4.00	17.33	17.92
40	*Symphoricarpos albus*	Caprifoliaceae	0.67	4.00	28.00	9.88
41	*Broussonetia papyrifera*	Urticaceae	4.00	1.33	23.00	20.00
42	*Cyperus rotundus*	Cyperaceae	0.67	4.67	7.00	13.25
43	*Rhodomyrtus tomentosa*	Myrtaceae	3.33	1.33	27.50	22.00
44	*Amaranthus tricolor*	Amaranthaceae	7.33	6.00	5.00	3.08
45	*Typha orientalis*	Typhaceae	2.00	6.00	9.25	14.13
46	*Ipomoea pes‐caprae*	Convolvulaceae	4.67	4.00	5.47	13.08
47	*Pharbitis purpurea*	Convolvulaceae	3.33	5.33	5.50	5.90
48	*Pollia japonica*	Commelinaceae	2.00	0.67	8.00	22.00
49	*Iris japonica*	Iridaceae	7.33	2.00	25.11	30.00
